# A Barcoded Polymer-Based Cross-Reactive Spectroscopic Sensor Array for Organic Volatiles

**DOI:** 10.3390/s19173683

**Published:** 2019-08-24

**Authors:** Jessica E. Fitzgerald, Jianliang Shen, Hicham Fenniri

**Affiliations:** 1 Department of Bioengineering, Northeastern University, Boston, MA 02115, USA; 2 School of Ophthalmology and Optometry, School of Biomedical Engineering, Wenzhou Medical University, Wenzhou 325000, China; 3 Wenzhou Institute, University of Chinese Academy of Sciences, Wenzhou 325000, China; 4 Department of Chemical Engineering, Northeastern University, Boston, MA 02115, USA; 5 Department of Chemistry & Chemical Biology, Northeastern University, Boston, MA 02115, USA

**Keywords:** barcoded polymers, sensor arrays, Raman spectroscopy, electronic nose

## Abstract

The development of cross-reactive sensor arrays for volatile organics (electronic noses, e-noses) is an active area of research. In this manuscript, we present a new format for barcoded polymer sensor arrays based on porous polymer beads. An array of nine self-encoded polymers was analyzed by Raman spectroscopy before and after exposure to a series of volatile organic compounds, and the changes in the vibrational fingerprints of their polymers was recorded before and after exposure. Our results show that the spectroscopic changes experienced by the porous spectroscopically encoded beads after exposure to an analyte can be used to identify and classify the target analytes. To expedite this analysis, analyte-specific changes induced in the sensor arrays were transformed into a response pattern using multivariate data analysis. These studies established the barcoded bead array format as a potentially effective sensing element in e-nose devices. Devices such as these have the potential to advance personalized medicine, providing a platform for non-invasive, real-time volatile metabolite detection.

## 1. Introduction

The design of artificial devices and systems using biomimetic engineering concepts emerged over three decades ago [1,2,3,4]. The scientific community recognized the power and versatility of natural sensing systems, such as the sense of smell, taste, touch, hearing, and vision, and began to mimic these natural functions, in particular for the design of so-called artificial/electronic noses (e-noses) [5,6]. Gardner and Bartlett defined an e-nose as a device composed of (a) an array of semi-selective sensing elements with a broad spectrum of specificities to a target analyte and (b) a pattern recognition system, capable of processing complex and often convoluted signal outputs and identifying unique pattern characteristic of a given analyte [5]. This technology platform was applied, for example, in the food industry [7,8,9], environmental monitoring [10], diagnostics [11,12,13,14,15,16,17,18,19,20,21,22], and public safety [23].

A hallmark of e-nose systems is cross-reactivity, i.e., no individual sensing element is selective toward an individual analyte. Instead, each sensing element could interact with several analytes with varying degrees of affinity, thereby resulting in a unique response pattern characteristic of the target analyte [6]. The resulting pattern of the cross-reactive sensor array (CRSA) can be used to not only classify but also quantify the analyte of concern [6,24,25,26,27,28,29,30,31].

E-nose devices can be classified on the basis of their signal-transduction mechanisms [29]. Among them, semiconducting metal oxides [32], conducting polymer films [24,33], acoustic wave devices [34,35,36,37], electrochemical systems [38,39,40], polymer film chemoresistors [22,25,41], and optical transducers [7,30,42,43,44,45,46] have been reported. Regardless of the transduction mechanism, the larger the number of sensory elements in a CRSA, the richer the data gathered, and the more specific the analyte identification and classification [47,48,49,50,51,52,53].

We have previously reported on a new class of polymers prepared from spectroscopically active styrene monomers, the combination of which produces polymers with unique vibrational fingerprints [49,51,54,55,56,57,58,59]. The spectrum from each polymer can then be converted into a barcode, in which the position of each bar matches a peak wavenumber in the spectrum. Each barcoded polymer (BP) can be identified with 100% confidence and classified using its unique vibrational spectra [58]. Recently, we have successfully demonstrated the ability of a BP thin film-based CRSA to identify analytes in the vapor phase [60] and in solution, including Hepatitis C coat protein p7 [61].

In this contribution, we have fabricated a CRSA using BP beads (BP-CRSAs) and tested the device for the detection and differentiation of clinically relevant volatile organic compounds (VOCs) as a first step towards developing a new assay for disease diagnosis. BP-CRSAs were fabricated by depositing an assortment of beaded BPs in an ordered fashion onto a glass microscope slide or quartz microscope coverslip inside a custom-built gas chamber. 

## 2. Materials and Methods

**Figure 1:**
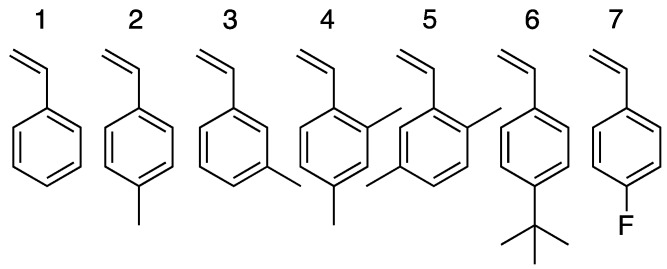
Molecular structure of the seven different monomers used for the preparation of the barcoded copolymer beads.

*Materials.* Polymer samples used in this paper were synthesized as previously described [55]. Nine polymers were selected from a library of ca. 700 to fabricate the BP-CRSAs. The polymers were made of a combination of seven monomers, as shown in Figure 1. Each polymer has a unique composition (supporting information) and vibrational fingerprint, which can be readily identified by Raman spectroscopy. 

Acetone, cumene, carbon disulfide (CS_2_), pentane, and propylbenzene (Aldrich, analytical-grade reagents used without further purification) were selected as target volatile organic analytes to test the effectiveness of the BP-CRSAs as an e-nose. These VOCs are known to be clinically relevant for disease diagnosis via exhaled breath analysis. Though they may also be present in the exhaled breath of patients with other diseases, the VOCs selected have the strongest association with the diseases in Table 1. This study will further establish that the sensors provide sufficient information for analyte classification.

**Figure 2:**
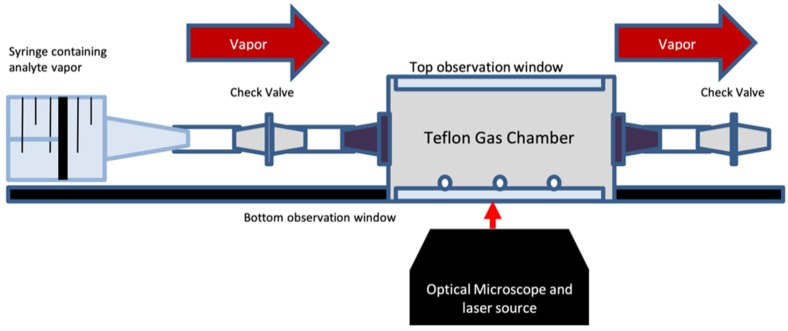
Vapor exposure setup using a custom-built gas chamber, micromachined from Teflon.

*Polymer sensor array fabrication.* Nine polymer bead types were selected from our library for the fabrication of each BP-CRSA. The beads were suspended in chloroform, and a small sample was spotted on quartz microscope coverslips (spot diameter ca. 1 mm, containing ca. 30 beads) contained within a custom-built gas chamber (Figure 2). Chloroform was chosen as an ideal solvent because of its low bowling point, weak Raman scattering, and because it promotes adhesion of the beads to the substrate while still allowing adequate exposure to analyte vapor. The beads did not show signs of wear or damage throughout the procedure.

*Experimental setup.* Prior to vapor exposure, the Raman spectra of each BP bead type were recorded in triplicate (control spectra). All experiments were performed at 20 °C and 50% relative humidity. Next, BP-CRSAs were placed in the head space of sealed glass bottles containing ca. 5 mL of VOC solution. After exposure to the analyte vapor for 5 min, the Raman spectra of the BP-CRSAs were recorded. For gas-chamber exposure (see illustration in Figure 2), the miniature gas chamber was sealed with a top window and inlet/outlet check valves, and then flushed with N_2_ gas for 90 s. Saturated analyte vapor collected from the head space of a solution of each analyte using a 30 mL gas syringe was then re-injected into the chamber through the inlet check valve. The vapor exposure method was designed to emulate the method used for administering breath samples in animal studies. The sensor arrays were scanned before and after analyte vapor exposure by Raman spectroscopy. After vapor exposure, the sensing device was allowed to equilibrate before spectrum or image acquisition. The equilibrium was verified by the absence of changes in the measured spectra, taken at two consecutive time points.

*Raman mapping of the BP-CRSAs.* The Raman spectra were obtained using a home-built inverted microscope at the Laser Biomedical Research Center (LBRC) at the Massachusetts Institute of Technology (see supporting information for details).

*Data processing.* The preprocessing of the vibrational spectra involved baseline correction and normalization. The spectral matrices before and after exposure were then compared by computing their dot products. This procedure was performed for each spectrum before and after VOC exposure (see supporting information for more details). An angle map was then generated, where *θ* is defined as the angle between the reference (unexposed) and response (exposed) vectors for each BP sensor [64,65,66] (see supporting information for details):

(1) $$\cos\theta =\frac{\left|y_{i}*y_{i}{}^{\prime }\right|}{\left|y_{i}\right|\left|y_{i}{}^{\prime }\right|}.$$

## 3. Results and Discussion

In this work, the fabrication and performance of a new sensor array for an e-nose device are presented. The fabrication techniques herein afford the fast, facile, cost-effective, and highly tunable production of microsensor arrays for the application of interest, compared to many published techniques for similar sensing arrays. The use of multivariate data analysis increases the sample’s throughput and processing time. Pattern recognition can then be used to identify the samples for which the vapor components and concentrations are unknown by referencing the library of known sensor responses for each unique BP. Batch to batch reproducibility and the sensitivity of CRSAs are cornerstones of e-nose technology. The ability of a sensor array to faithfully recognize an analyte is essential for any application. Sensitivity here is defined as the degree of response (*θ*) for each BP, relative to the analyte concentration. This was evaluated for CS_2_ and was chosen because of its strong and consistent spectral response fingerprint for all BPs. The results are presented below. 

*Microarray fabrication and testing.* From an initial library of ca. 700 BPs, each with a unique vibrational barcode [57], nine BPs (formulations 14 through 22) were selected for the fabrication of each BP-CRSA. The monomers used to synthesize the BPs are presented in Figure 1. Inspection of the arrays by optical microscopy confirmed that, after sensor fabrication, the beads did not show signs of wear or damage. Using the bead deposition procedure described in the experimental section, it was possible to fabricate arrays containing either one type of bead for a single polymer response analysis, or a selection of BPs, for a global sensor response analysis.

Raman spectroscopy of each microspot containing beads from a single class of BPs enables accurate and quick identification of each BP class in the microsensor array. The BP beads placed in each microarray can be distinguished from one another through their unique Raman spectra. The identification and testing of the polymer bead arrays were performed using a Raman microscope equipped with a homemade miniaturized gas chamber (Figure 2). This device allows for the addition of a VOC via a syringe through an inlet check valve. The miniaturized gas chamber itself provides excellent resistance to aggressive organic solvents and allows the preparation of BP bead arrays for Raman analysis. Because of its excellent durability with respect to the solvent, the support can be deconstructed and then resealed with a fresh array of sensors and reused many times without cross-contamination.

**Figure 3:**
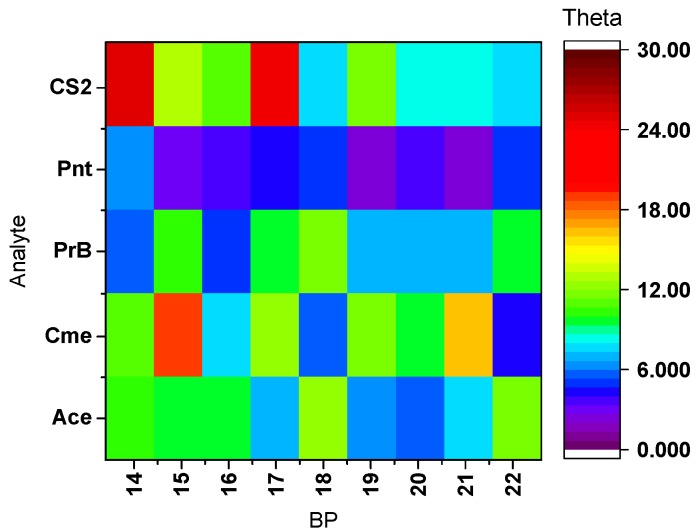
A theta heat map of polymer/analyte interactions compared to the control (Ctr). Analytes studied: acetone (Ace), cumene (Cme), propylbenzene (PrB), pentane (Pnt), and carbon disulfide (CS2).

*Multivariate data analysis and sensor arrays response.* All of the analytes produced unique changes in the initial Raman spectra of the BPs in the BP-CRSA (supporting information). These changes likely arise from multiple interactions due to physisorption and/or chemisorption of the analyte on the polymer matrices. The θ heat map for each analyte (Figure 3) clearly shows a unique and reproducible [67] pattern response that can be used for their rapid identification. To validate the consistency of the control spectra for each BP between scans, the θ values between all three control runs (before exposure) were calculated.

*Reproducibility.* As in biological systems, an e-nose is a device that can be trained to recognize an ever-increasing set of analytes as long as each analyte results in a unique response pattern. As this database expands, the ability of a CRSA to identify, classify, and possibly quantify an analyte becomes not only possible but also much more accurate. Furthermore, natural and artificial sensors have a finite lifetime. For example, olfactory receptor cells are constantly regenerated and are believed to have a lifespan of 4–8 weeks [68], yet, once a natural system is exposed to an “odor” for the first time, it will always recognize that odor. In the present study, the high degree of sensor reproducibility coupled with the simple fabrication and low cost of the sensor arrays makes the periodic replacement of the entire array simple and cost-effective and would not require retraining the arrays.

**Figure 4:**
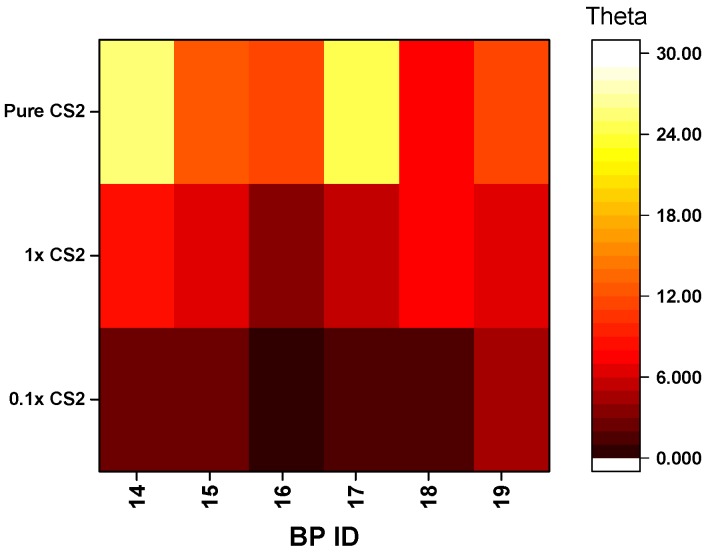
Stepwise increase of theta values upon increasing the concentration of CS_2_ for six of the barcoded polymers (BPs).

*Sensitivity of the BP-CRSAs.* To evaluate the detection limit of the BP-CRSAs, various concentrations of CS_2_ in water were prepared, and the solvent vapor headspace was then injected into the gas chamber. Given that a saturated aqueous solution of CS_2_ contains 2.17 g/L at 20 °C, we prepared two concentrations at 100% and 10% saturation (2.17 g/L and 0.217 g/L, respectively), recorded the spectra, and calculated the BP-CRSA responses. Compared with pure CS_2_ headspace vapor, responses for the dilute CS_2_ solutions were reduced, as shown in Figure 4. 

## 4. Conclusions

Cross-reactive polymer microbead arrays were fabricated. Using Raman spectroscopy, the arrays can be readily scanned, and the identity of each polymers can be determined by correlating the chemometric image with the known spectra of the polymers used. Arrays of these BPs were shown to be effective sensors for biologically relevant analytes. However, analyte concentrations in exhaled breath are several times lower than the concentrations tested (parts per million to parts per billion). Thus, further testing and optimization are needed to boost the sensor sensitivity and reproducibility for clinical applications using exhaled human breath. To realize this aim, we plan to modify our BP sensor beads by incorporating surface enhanced Raman scattering (SERS) technology, such as that used by our group for the improved detection of serotonin using optically stable gold-polyethylene glycol-polystyrene nanocomposites [69] or our silver nanoparticle-polystyrene bead composites [52]. SERS has been shown to increase sensitivity by several orders of magnitude [70]. SERS, then, can significantly increase the potential of the BP arrays for implementation in clinical applications, including disease detection and diagnosis in exhaled breath.

## Figures and Tables

**Table 1:** List of diseases and their associated volatile organic compounds (VOCs).

Diseases	Associated VOCs
Alzheimer’s disease	Propyl-benzene, 1-Methylethyl-benzene (Cumene) [19]
Schizophrenia	Pentane, Carbon Disulfide [62]
Diabetes	Acetone [63]

## Supplementary Materials

The following are available online at https://www.mdpi.com/1424-8220/19/17/3683/s1, Figure S1: Raman spectra of BPs before and after exposure to analytes, Table S1: BP composition.
