# Supplementary material for: A Barcoded Polymer-Based Cross-Reactive Spectroscopic Sensor Array for Organic Volatiles
Source: Sensors (Basel). 2019 Aug 24;19(17):3683. doi: 10.3390/s19173683 (PMC6749357; doi:10.3390/s19173683)
Supplement: Supplementary file 1 [file sensors-19-03683-s001.pdf]

Supporting Information for:

Barcoded Polymer-Based Cross-Reactive Spectroscopic Sensor Array for Organic Volatiles

Jessica Fitzgerald[1], Jianliang Shen[2] and Hicham Fenniri[1,3]*

[1] Department of Bioengineering, Northeastern University, Boston, MA 02115, USA; fitzgerald.je@husky.neu.edu
[2] School of Ophthalmology and Optometry, School of Biomedical Engineering, Wenzhou Medical University, Wenzhou, China; Wenzhou Institute, University of Chinese Academy of Sciences, Wenzhou, China; shenjl@wibe.ac.cn
[3] Department of Chemical Engineering, Northeastern University, Boston, MA 02115, USA; Department of Chemistry & Chemical Biology, Northeastern University, Boston, MA 02115, USA; h.fenniri@northeastern.edu
* Correspondence: h.fenniri@northeastern.edu; Tel.: +1-617-373-7690.

Raman mapping of BP-CRSAs. The Raman spectra were obtained using a home-built inverted microscope at the Laser Biomedical Research Center (LBRC) at the Massachusetts Institute of Technology (MIT). Variable intensity-controlled white LEDs was used as an illumination source for bright field imaging. The CMOS camera (BCN-B050-U, MightTex) was used to capture the image. A frequency-doubled Nd:YAG laser (Millennia 5sJ, Spectra-Physics) at 532 nm was the pump source for a CW tunable Ti:sapphire Laser (3900S, Spectra-Physics). The 785 nm output was used as the excitation source for both the confocal reflectance and confocal Raman systems. For high-resolution imaging, a water immersion objective lens (Olympus UPLSAPO60XWIR 60X/1.20) was used. A flip mirror, placed after the tube lens, allows the image focused at the sample plane from the incoherent transmission source to be observed at the video camera with *ca.*67× magnification. The collimated beam is band pass filtered (BPF, LL01-785-12.5, Semrock) and redirected to dual-axis galvanometer mirrors (CT-6210, Cambridge Technology) by a dichroic mirror (DM1, LPD01-785RU-25, Semrock). After the galvanometer mirrors, the beam size was adjusted by a telescope and focused at the sample plane by an objective lens. X-Y positioning was achieved by a micrometer-controlled stage (406, New Focus). Coarse and fine adjustment of sample focus was done using a piezo-actuator combined with a differential micrometer (DRV517, Thorlabs). The spectra were recorded in the range of 178-1668 cm^{-1}. For each analyte and the control (non-exposed), spectra were collected with an exposure time of 1 s with 9 scans accumulation. For each analyte exposure experiment, 3 separate beads of each BP were scanned to evaluate reproducibility of the sensor response. The averages of the accumulated spectra of the 3 beads for each BP was then used for comparison and analysis (3 sets of spectra for 9 different BP classes in total).

Data processing. All spectra of the BP sensors before and after exposure were imported into the Unscrambler software (Camo). All raw spectra were plotted and filtered to eliminate any interference from γ-rays. Spectra were then normalized to 1 and the baseline subtracted using the second derivative function in Origin software. The corrected spectra were then added to the Unscrambler file, smoothed using a 5-point Savitzy-Golay algorithm, and the first derivative was taken to eliminate any further discrepancies in the baseline between spectra.

The spectral matrix is a collection of vectors for each BP, each with a unique spectral vector in 985-dimensional space, y_i, where each dimension represents a relative intensity for any given wavenumber. To compare the sensor arrays' responses to the analytes, the vectors obtained from the spectra matrix before and after the analyte vapor treatment were considered the "reference" and "response" matrices, respectively. For a given trial, 9 BPs beads were used to construct a sensor array, and thus, spectral data was represented as a spectral matrix of reference and response scans (Y and Y′, respectively) with dimensions 9×985. An angle map was then generated where θ is defined as the angle between the reference and response vectors for each BP sensor. The angle between every y_i and y_i' was determined by the inner product of the two vectors [65-67]:

$$cos\theta = \frac{|y_i * y_i'|}{|y_i||y_i'|} \quad \text{(Equation 1)}$$

This gave 81 (9×9) vector pairings or angles. From equation 1, it can be seen that if $y_i = y_i'$ then $\cos\theta = 1$, thus $\theta = 0$. This shows that the closer the response vectors are to the reference vectors, the smaller the angle between y_i and y_i' will be. Moreover, by comparing vectors before (reference) and after (response) the interaction of the BP beads with the analyte vapor, a response pattern could be generated for each analyte. Therefore, the angle would reflect the degree of spectral change of each BP sensor due to the analyte vapor exposure.

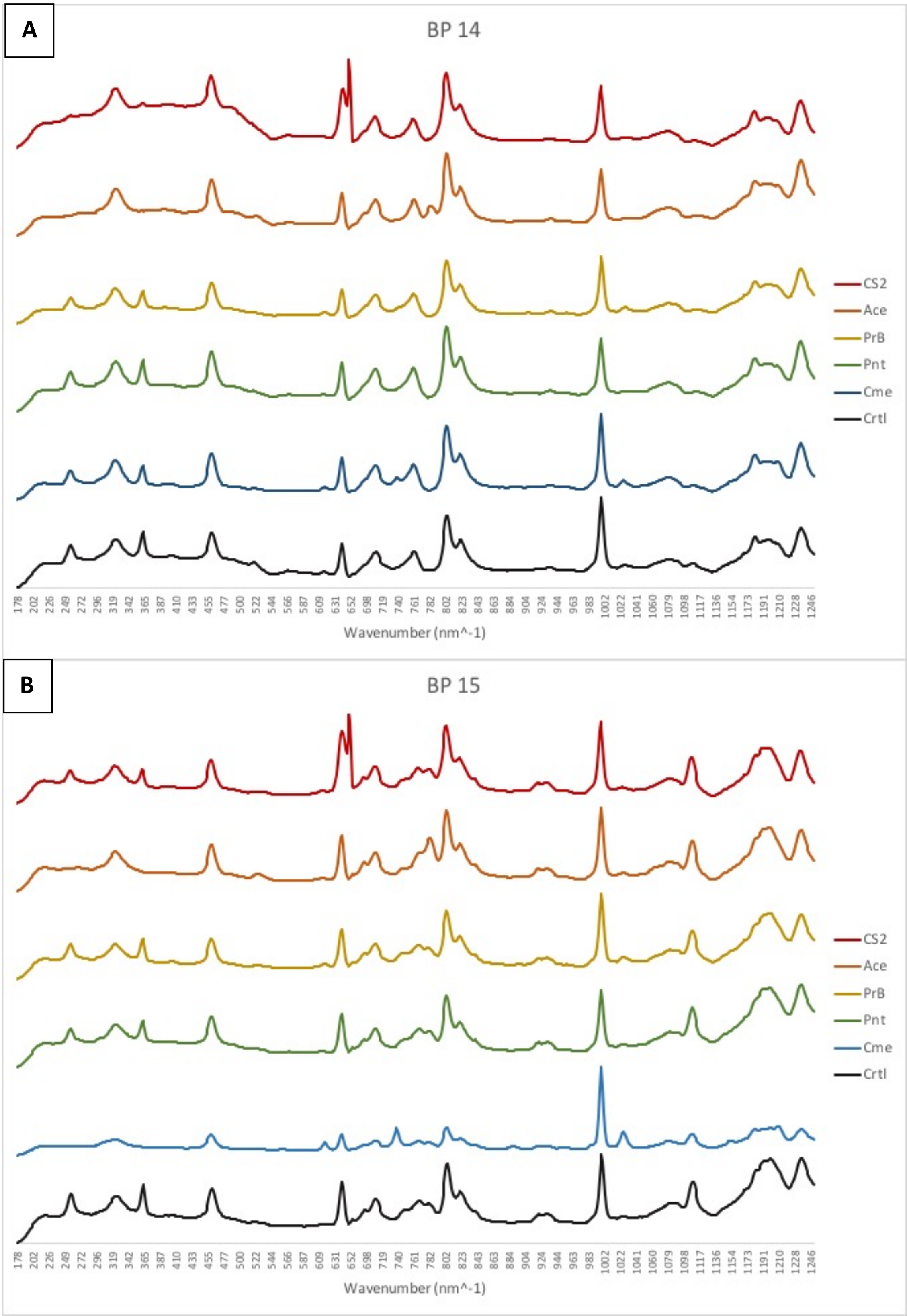
A
BP 14
CS2
Ace
PrB
Pnt
Cme
Crtl
178 202 226 249 272 296 319 342 365 387 410 433 455 477 500 522 544 566 587 609 631 652 698 719 740 761 782 802 823 843 863 884 904 924 944 963 983 1002 1022 1041 1060 1079 1098 1117 1136 1154 1173 1191 1210 1228 1246
Wavenumber (nm^-1)
B
BP 15
CS2
Ace
PrB
Pnt
Cme
Crtl
178 202 226 249 272 296 319 342 365 387 410 433 455 477 500 522 544 566 587 609 631 652 698 719 740 761 782 802 823 843 863 884 904 924 944 963 983 1002 1022 1041 1060 1079 1098 1117 1136 1154 1173 1191 1210 1228 1246
Wavenumber (nm^-1)

C

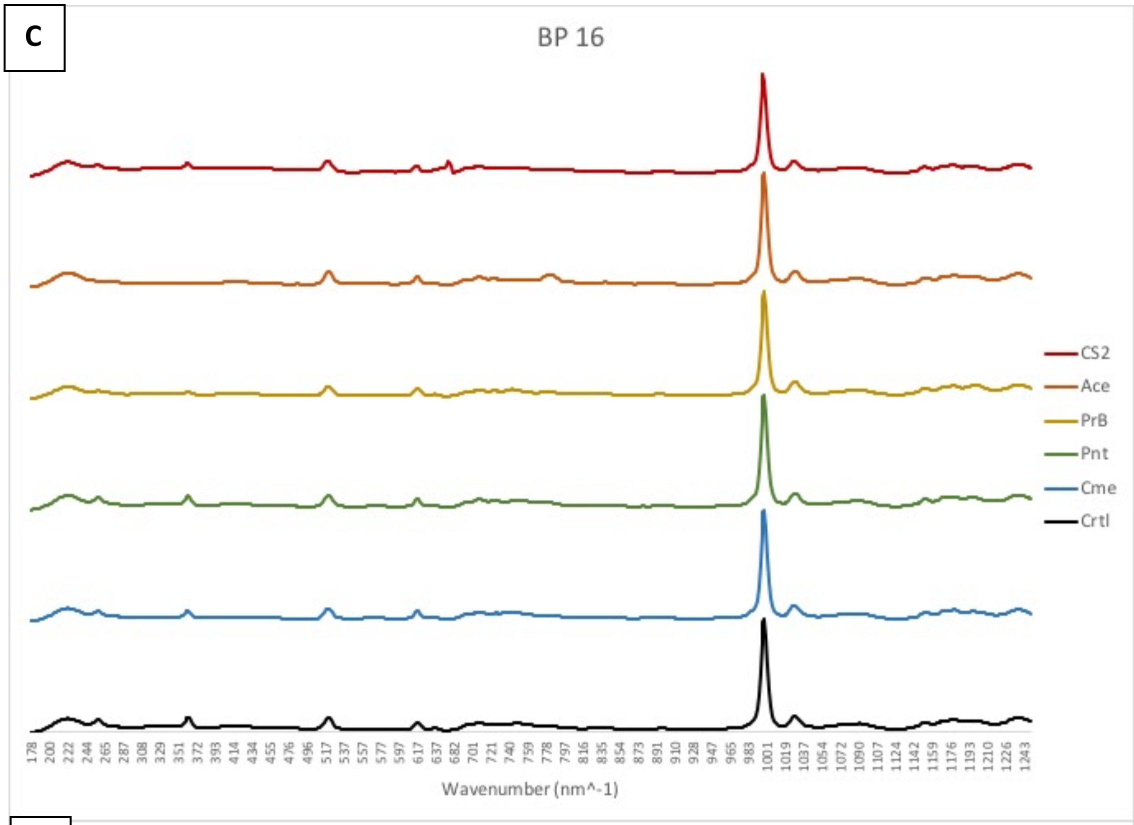
BP 16
CS2
Ace
PrB
Pnt
Cme
Crtl
Wavenumber (nm^-1)

D

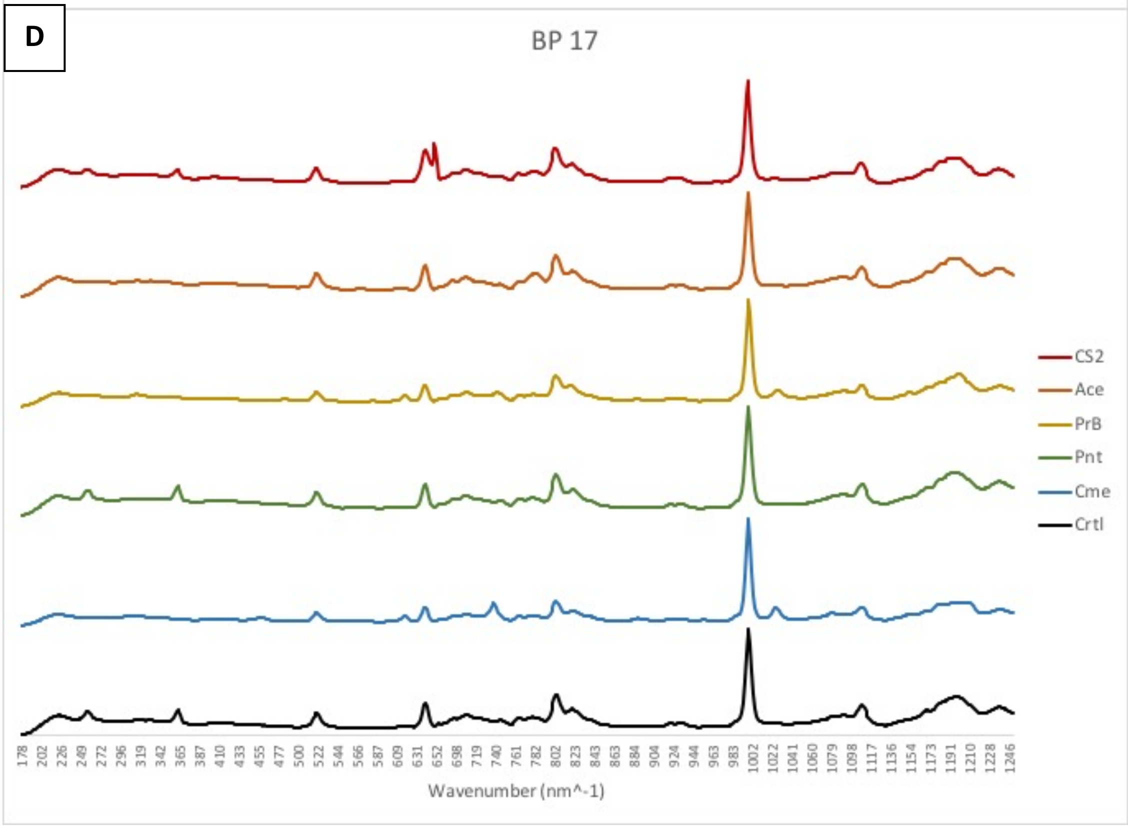
BP 17
CS2
Ace
PrB
Pnt
Cme
Crtl
Wavenumber (nm^-1)

E

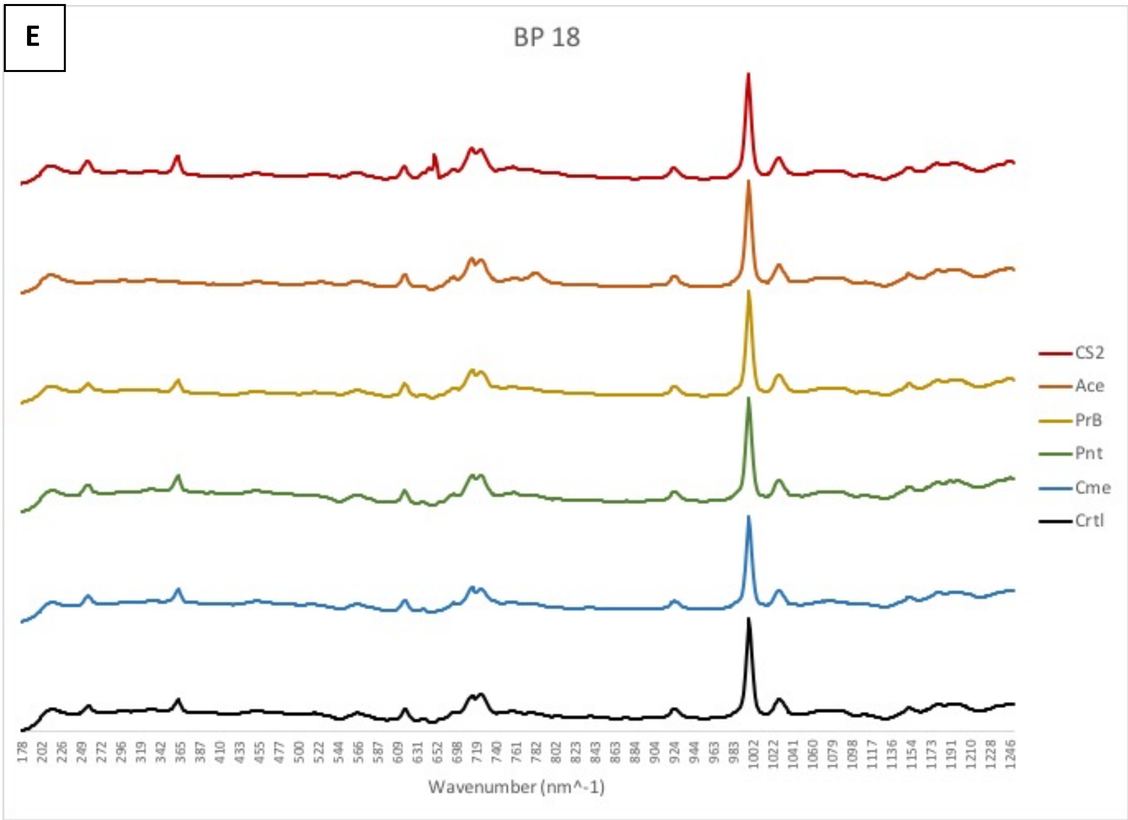
BP 18
CS2
Ace
PrB
Pnt
Cme
Crtl
Wavenumber (nm^-1)

F

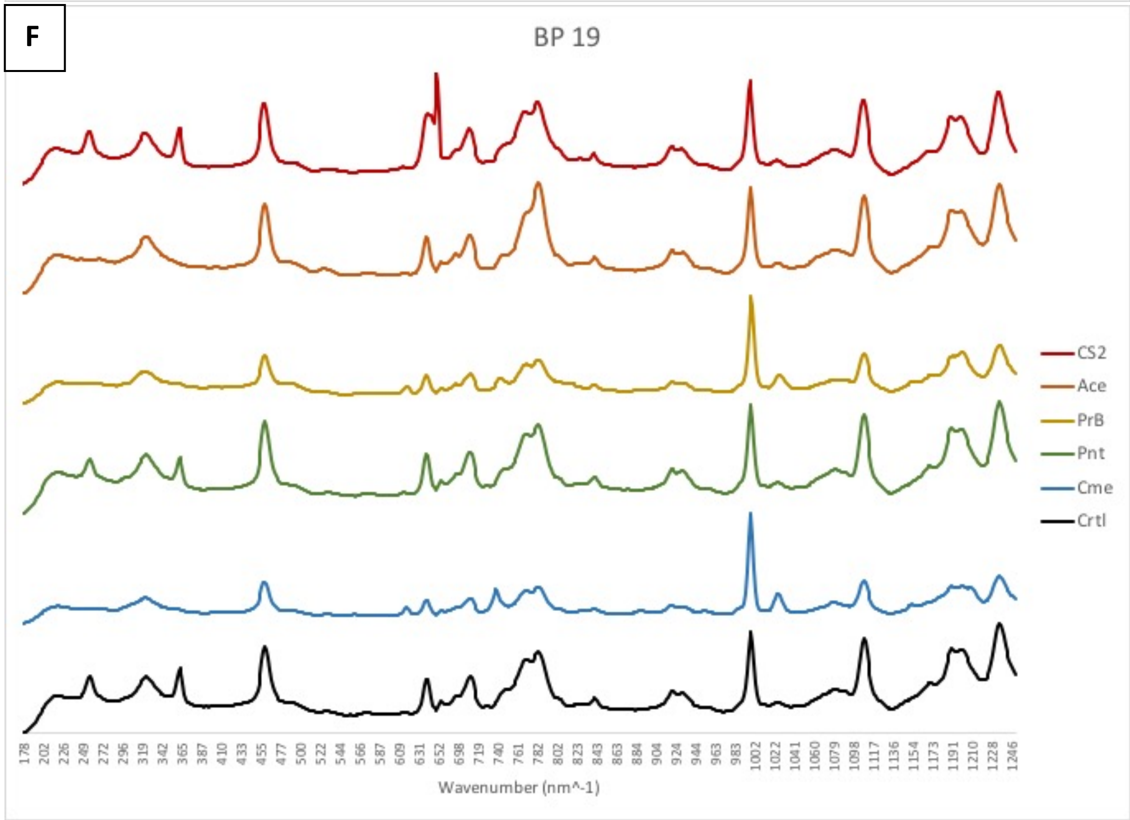
BP 19
CS2
Ace
PrB
Pnt
Cme
Crtl
Wavenumber (nm^-1)

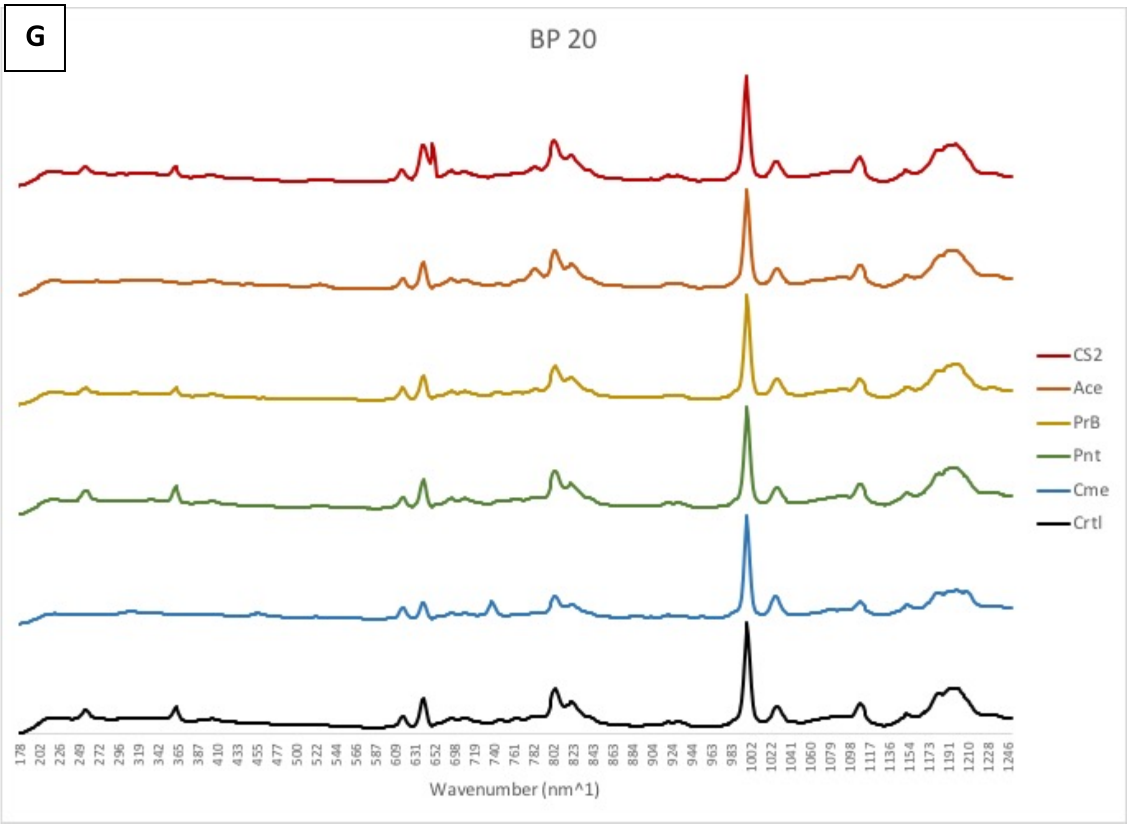
G
BP 20
CS2
Ace
PrB
Pnt
Cme
Crtl
Wavenumber (nm^1)

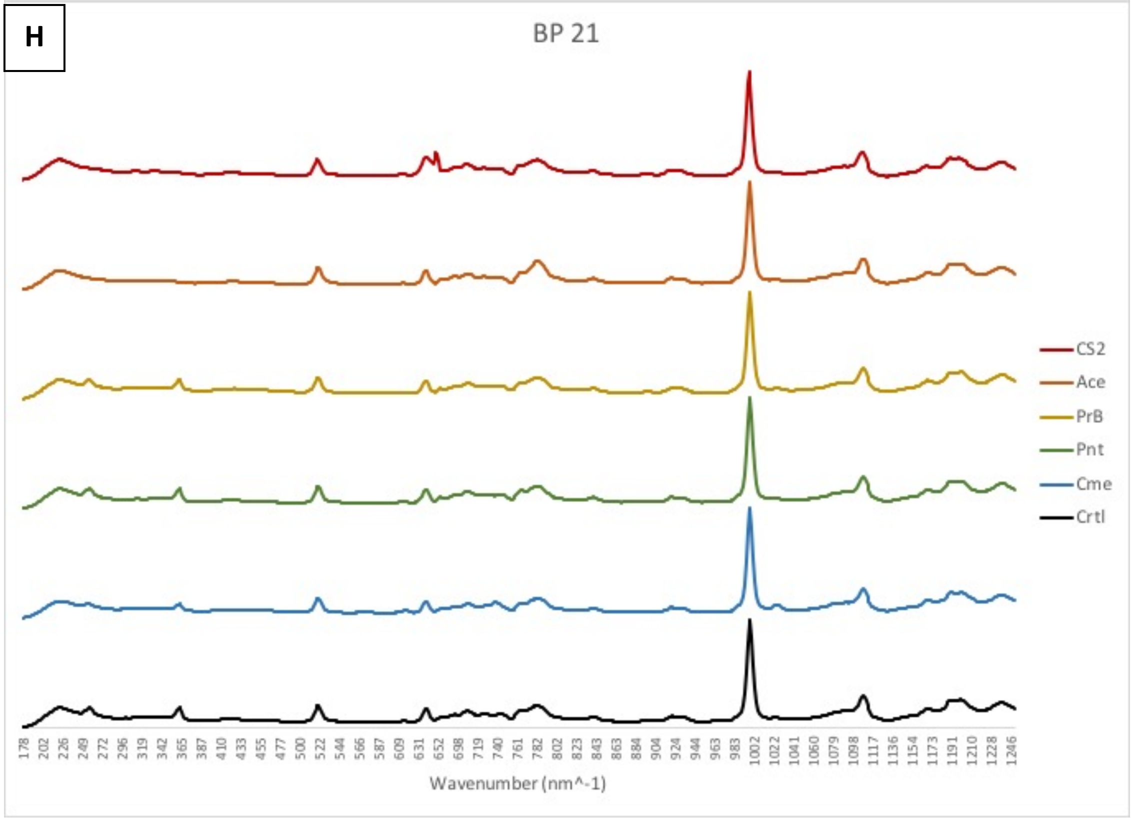
H
BP 21
CS2
Ace
PrB
Pnt
Cme
Crtl
Wavenumber (nm^-1)

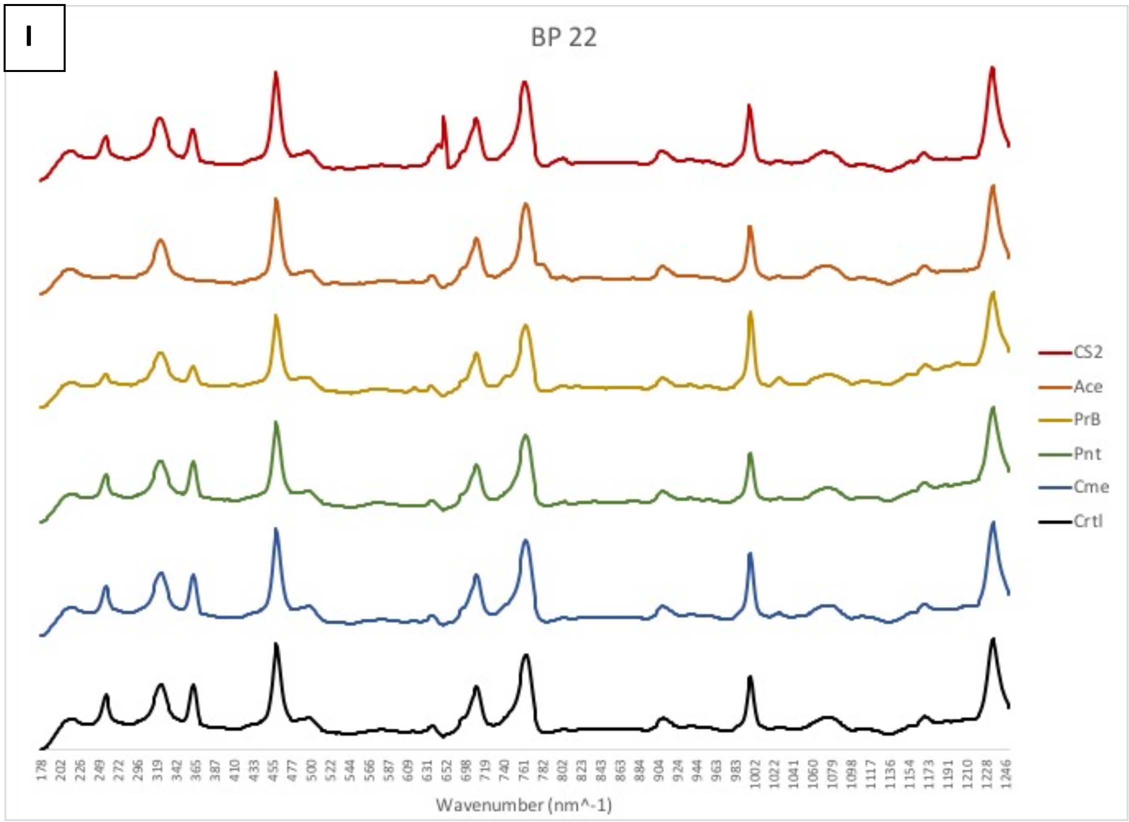

Figure S1. Raman spectra of BPs before and after exposure to analytes. Raman spectra of each BP before (control) and after analyte exposure from 178-1246 nm^{-1}. Spectra from top to bottom in each plot: carbon disulfide (CS2; red), acetone (Ace; orange), propyl benzene (PrB; yellow), pentane (Pnt; green), cumene (Cme; blue), control (Ctr; black). A) BP 14, B) BP 15, C) BP 16, D) BP 17, E) BP 18, F) BP 19, G) BP 20, H) BP 21, I) BP 22.

Table S1. BP composition (wt%).

**Monomer→**	**1**	**5**	**2**	**4**	**6**	**3**
**BP bead↓**						
**14**	0.000	50.000	50.000	0.000	0.000	0.000
**15**	0.000	33.333	33.333	0.000	33.333	0.000
**16**	50.000	0.000	0.000	0.000	0.000	50.000
**17**	0.000	0.000	33.333	0.000	33.333	33.333
**18**	50.000	0.000	0.000	50.000	0.000	0.000
**19**	0.000	50.000	0.000	0.000	50.000	0.000
**20**	33.333	0.000	33.333	0.000	33.333	0.000
**21**	0.000	0.000	0.000	0.000	50.000	50.000
**22**	0.000	100.000	0.000	0.000	0.000	0.000
